# 3-Hydroxyflavone Inhibits Biofilm Formation and Enhances Antibiotic Activity Against *Enterococcus faecalis* and *Enterococcus faecium*

**DOI:** 10.3390/antibiotics15090925

**Published:** 2026-09-18

**Authors:** Ji-Hyun Yoon, Yeo-Jin Kim, Ki-Young Kim

**Affiliations:** 1 Graduate School of Biotechnology, Kyung Hee University, Yongin 17104, Republic of Korea; hayop0909@khu.ac.kr; 2 Major in Bio-Cosmetic Engineering, Division of Convergence Studies, Sungkyul University, Anyang 14097, Republic of Korea

**Keywords:** 3-Hydroxyflavone, bacterial biofilm, oral bacteria, antibiotic adjuvants, antibacterial resistance

## Abstract

**Background/Objectives:** The increasing antibiotic resistance of pathogenic bacteria has created an urgent need for new therapies that target virulence factors. Biofilm formation is a major determinant of bacterial pathogenicity and antimicrobial resistance. **Methods:** Biofilm formation assays (using crystal violet staining) were performed in independent triplicates to evaluate the biofilm-inhibitory activity of 3-Hydroxyflavone. A bacterial viability assay (colony-forming unit counting) was used to assess the enhanced antibiotic activity, and qRT-PCR analysis was conducted to investigate the transcriptional modulation of biofilm-related genes. **Results:** 3-Hydroxyflavone inhibited biofilm formation in *E. faecalis* (IC_50_ = 1.025 μg/mL), *E. faecium* (IC_50_ = 0.19 μg/mL), *S. aureus* (IC_50_ = 1.21 μg/mL), *C. acnes* (IC_50_ = 0.132 μg/mL), *S. sobrinus* (IC_50_ = 5.49 μg/mL), *P. aeruginosa* (IC_50_ = 22.1 μg/mL), and *E. coli* (IC_50_ = 10.88 μg/mL). Notably, these effects were observed without significant inhibition of planktonic bacterial growth, indicating a biofilm-specific mechanism of action. 3-Hydroxyflavone also downregulated the expression of quorum-sensing genes (*FsrB*, *FsrC*, *GelE*, *EbpA*, *EbpB*, *Acm*, *Scm*, and *Bps*), the biofilm virulence gene *Esp*, and cytolysin genes (*CylLs*, *CylR*, and *CylM*), while exhibiting strong enhanced antibiotic activity in the viability assay. **Conclusions:** These in vitro findings suggest that 3-Hydroxyflavone may serve as a promising antibacterial adjuvant for controlling biofilm-associated bacterial infections. However, further studies, including in vivo validation, are required to determine its clinical applicability.

## 1. Introduction

Bacterial biofilms are structured communities of bacterial cells embedded within a self-produced extracellular matrix that adheres to the host surface [1]. Biofilms present a major challenge in medicine, industry, and dentistry because their specialized architecture enhances survival under environmental stress, including exposure to antibiotics and host immune responses [2]. Consequently, biofilm formation is a relevant target in the development of antibacterial adjuvants [3,4,5].

Oral bacteria predominantly reside in biofilms or dental plaque, where microbial communities adhere to oral surfaces and are encased within an extracellular polymeric matrix that promotes structural stability and coordinated interspecies interactions. Oral biofilms are highly organized multispecies microbial consortia. Among these, *Enterococcus* spp., particularly *Enterococcus faecalis* and *Enterococcus faecium*, are notorious opportunists [6]. We focused on these two species because they exhibit robust biofilm-forming capacity, possess intrinsic and acquired multidrug resistance, and contribute significantly to the persistence and pathogenicity of chronic oral infections, including dental caries, endodontic disease, periodontitis, and peri-implantitis. Owing to their enhanced adhesion and increased tolerance to antimicrobial agents and host immune defenses, enterococcal populations within oral biofilms are strongly associated with therapeutic failure [5,6,7,8,9,10,11,12,13,14].

Biofilm-associated genes are valuable for predicting the virulence potential of clinical enterococcal isolates and their contribution to enterococcal infections [15]. In *E. faecalis* and *E. faecium*, the endocarditis- and biofilm-associated pilus (Ebp) locus comprises the structural pilin genes *EbpA*, *EbpB*, and *EbpC*, along with the adjacent *Bps* gene, which encodes a class C sortase required for pilus polymerization and surface display, respectively. These pili promote adhesion, biofilm formation on abiotic surfaces, and virulence in experimental models of endocarditis and urinary tract infection. Importantly, because *E. faecalis* and *E. faecium* are versatile pathogens capable of translocating from oral reservoirs to cause severe systemic conditions, including urinary tract infections (UTIs), the human urinary bladder carcinoma T24 cell line was employed in this study as an established and highly relevant in vitro model to evaluate bacterial host–cell adhesion. In addition, the enterococcal surface protein Esp is an important determinant of biofilm development in *E. faecalis* and *E. faecium* [16]. Collectively, multiple genes and gene clusters have been implicated in enterococcal biofilm formation, underscoring the multicomponent and genetically complex nature of this phenotype [17,18,19,20,21,22].

**Figure 1:**
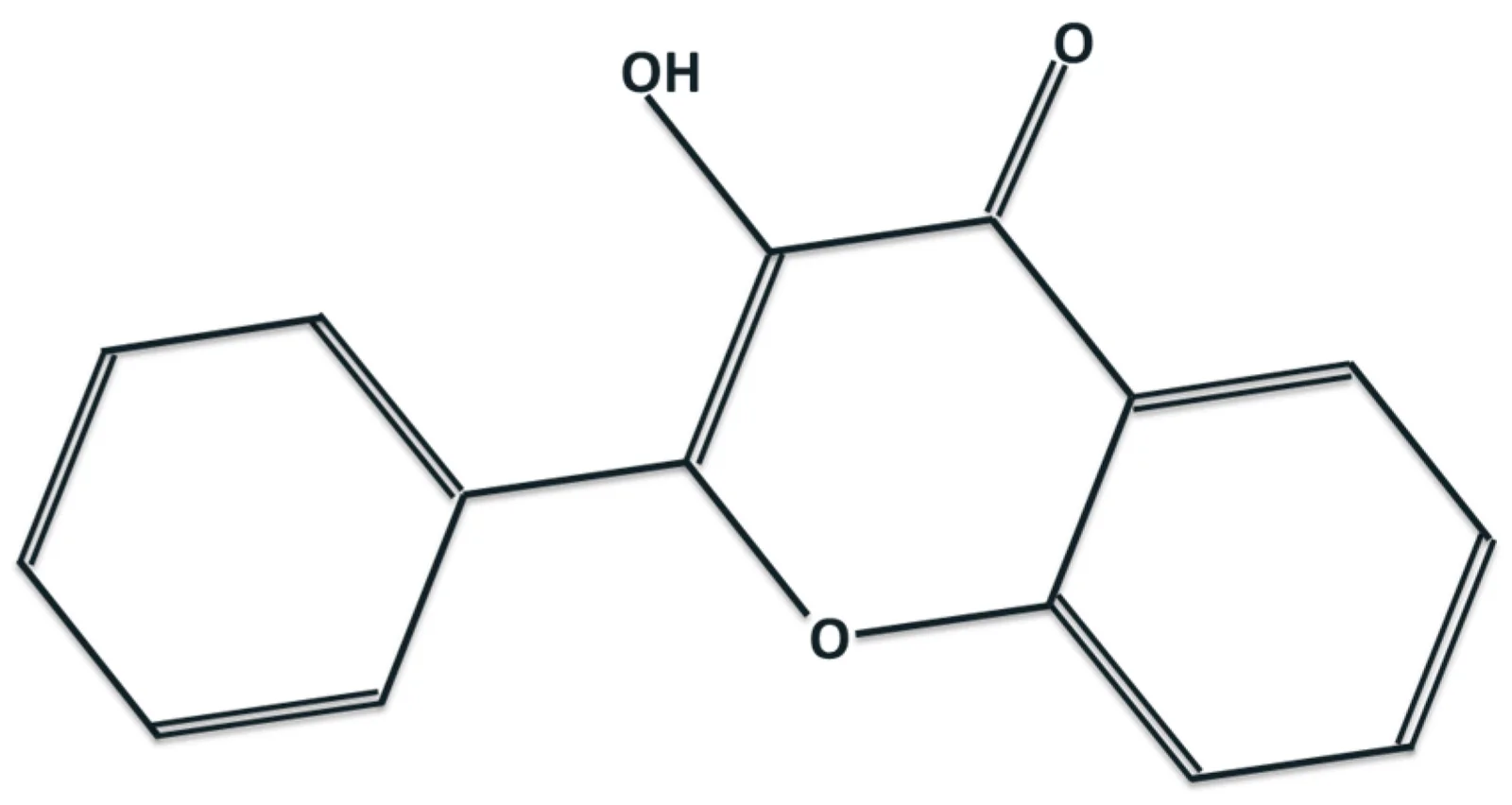
Chemical structure of 3-Hydroxyflavone.

3-Hydroxyflavone (3HF) (Figure 1), also known as flavon-3-ol, is the core scaffold of flavonols, a major subclass of flavonoids widely distributed in plants and recognized for their diverse biological activities [23,24]. Structurally, 3HF contains a 3-hydroxyl group conjugated with the 4-carbonyl moiety and the 2,3-double bond, which confers an extended *π*-electron system, strong metal-chelating potential, and pronounced fluorescence properties associated with excited-state intramolecular proton transfer [25].

Owing to these physicochemical features, 3HF and its derivatives have attracted considerable attention in medicinal chemistry, chemical biology, and materials science. Even though reported to exhibit antioxidant, anti-inflammatory, anticancer, antitumor, antiviral, and anticholinesterase effects, and synthetic modifications of the 3HF scaffold have been pursued to improve potency, selectivity, and pharmacological performance [19,26], no studies have reported the antibacterial effects of 3HF. To discover novel antibiofilm agents, we initially screened 14 structurally diverse flavonoid compounds, leading to the selection of 3HF as the most potent candidate [27].

In this study, 3HF was evaluated for its ability to inhibit biofilm formation by pathogenic bacteria, with the aim of assessing its potential as an antibacterial adjuvant candidate. We hypothesized that 3HF would effectively disrupt enterococcal quorum-sensing networks, thereby inhibiting biofilm formation, reducing host–cell adhesion, and ultimately enhancing the susceptibility of these multidrug-resistant pathogens to conventional antibiotics.

## 2. Results

### 2.1. 3HF Exhibited Strong Biofilm Formation Inhibitory Activity Against E. faecalis

Biofilms are important for bacterial survival and infections. Previous studies have shown that flavonoids have anti-biofilm formation activity against *Pseudomonas aeruginosa* and *Staphylococcus aureus* [17,18]. Therefore, we initially tested the antibiofilm activity of 14 flavonoid compounds against *E. faecalis* and found that only 3HF showed high inhibitory activity (Table 1).

**Figure 2:**
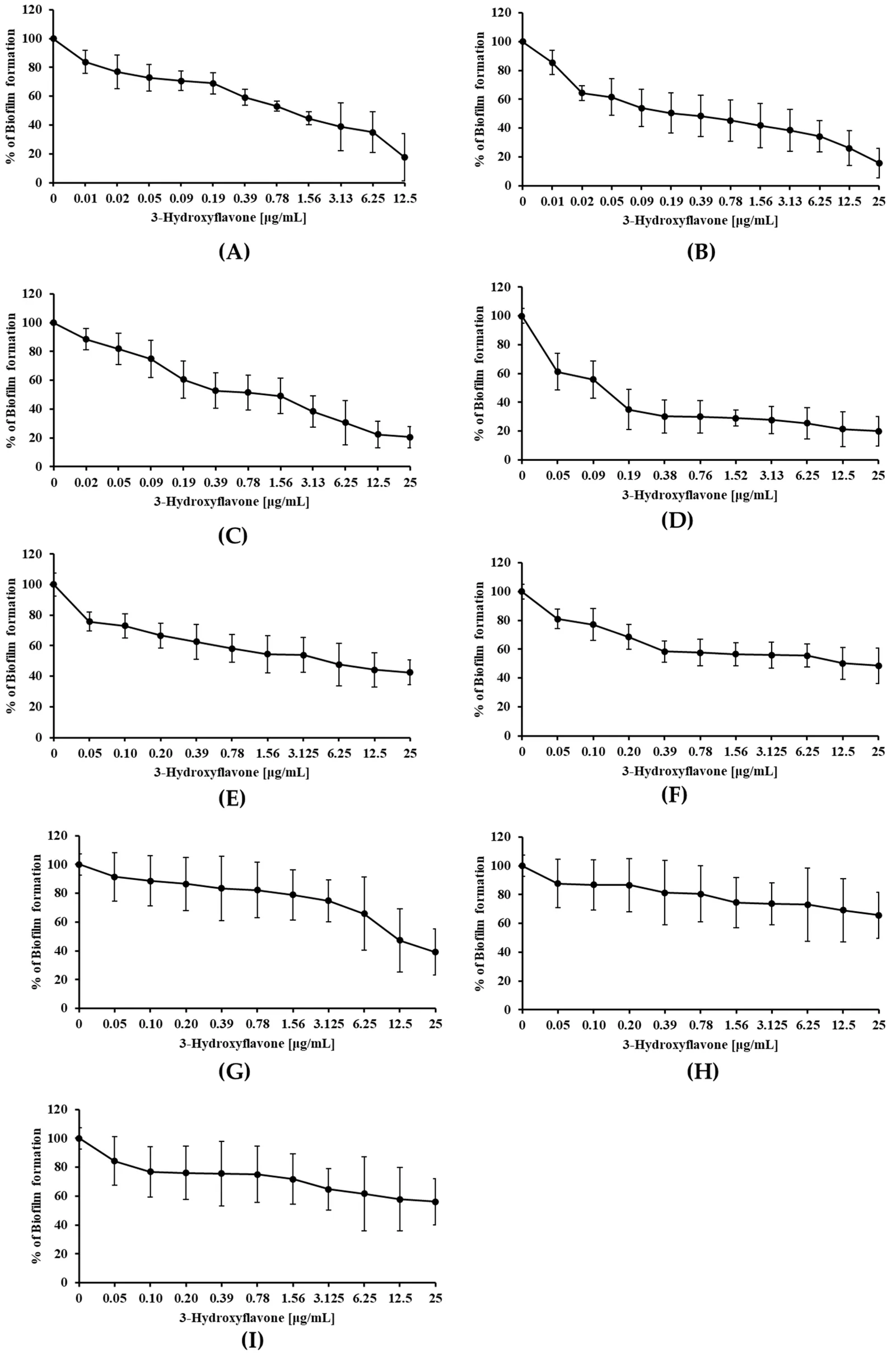
Inhibitory effects of 3HF on biofilm formation in (**A**) *E. faecalis*, (**B**) *E. faecium*, (**C**) *S. aureus*, (**D**) *C. acnes*, (**E**) *S. sobrinus*, (**F**) *P. aeruginosa*, (**G**) *E. coli*, (**H**) *P. gingivalis*, and (**I**) *S. mutans*. Data are presented as mean ± SD (n = 3).

Based on this, we further tested the anti-biofilm formation activity of 3HF and found dose-dependent inhibition of the biofilm formation of *E. faecalis* (IC_50_ = 1.025 μg/mL), *E. faecium* (IC_50_ = 0.19 μg/mL), *S. aureus* (IC_50_ = 1.21 μg/mL), *Cutibacterium acnes* (IC_50_ = 0.132 μg/mL), *Streptococcus sobrinus* (IC_50_ = 5.49 μg/mL), *P. aeruginosa* (IC_50_ = 22.1 μg/mL), and *Escherichia coli* (IC_50_ = 10.88 μg/mL). 3HF showed a low biofilm formation inhibitory effect on *Porphyromonas gingivalis* and *Streptococcus mutans* at the tested concentrations (Figure 2 and Table 2).

3HF suppressed biofilm formation in *E. faecalis* and *E. faecium* more than in the other bacterial species tested. Prior to defining these strains as resistant, we confirmed their antimicrobial resistance profiles; both *E. faecalis* (CCARM 5511) and *E. faecium* (KACC 11954) exhibited substantial tolerance to several conventional antibiotics, including vancomycin and ampicillin. Therefore, these clinically important, drug-resistant *Enterococci* were selected for further study.

### 2.2. 3HF Increased the Susceptibility of E. faecalis and E. faecium to Clinically Used Antibiotics

**Figure 3:**
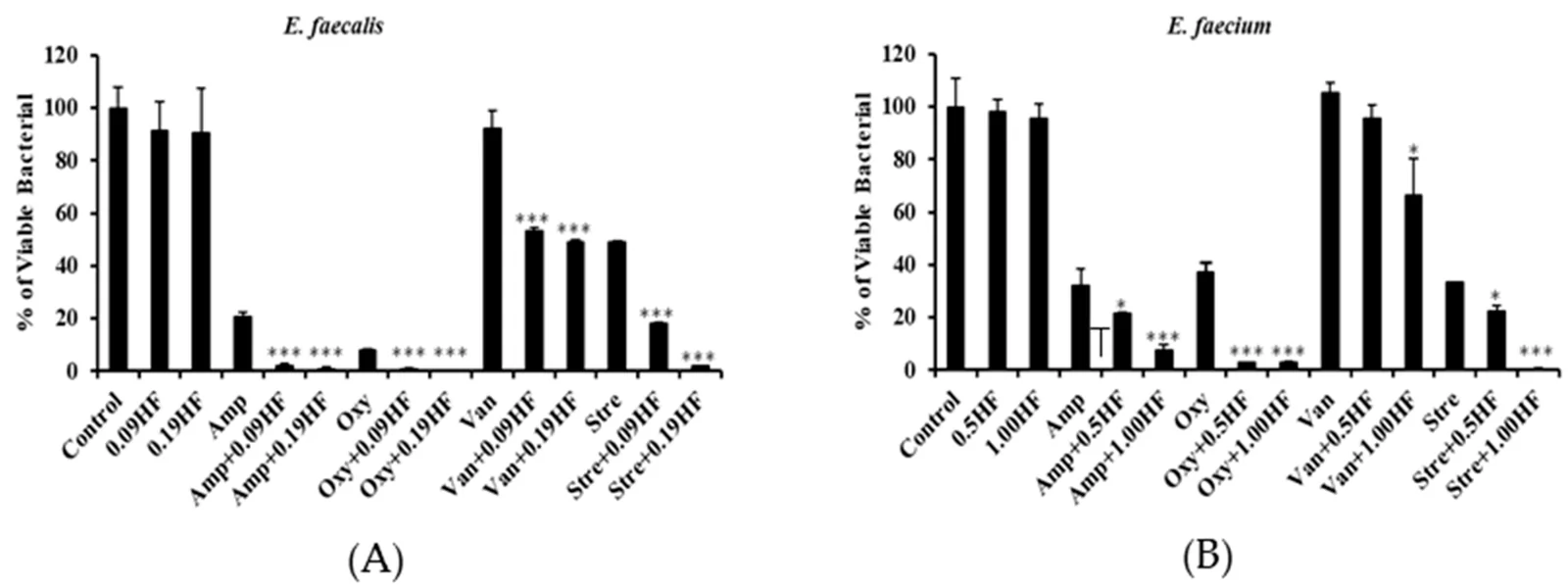
3HF enhances antibiotic susceptibility in (**A**) *E. faecalis* and (**B**) *E. faecium*. Data are presented as mean ± SD (n = 3). Statistical comparisons were made against the respective antibiotic-only treatment groups (* *p* < 0.05, *** *p* < 0.001).

Antibiotics are commonly used to control the growth of *E. faecalis* and *E. faecium*, and bacterial biofilms contribute to bacterial tolerance to these agents. Biofilms were formed as described above, and bacterial viability was assessed after an additional 24 h of treatment with ampicillin (AMP), vancomycin (VAN), oxytetracycline (OXY), or streptomycin (STR), either alone or in combination with 3HF. 3HF significantly enhanced antibiotic susceptibility, regardless of strain resistance to antibiotics (Figure 3). In *E. faecalis*, treatment with 0.19 μg/mL 3HF significantly enhanced the antibacterial activity of the antibiotics, reducing viable cells to 0.93% compared to 20.81% with ampicillin alone and 0.41% compared to 7.9% with oxytetracycline alone. Notably, 0.19 μg/mL 3HF reduced bacterial viability by 49.15% in combination with vancomycin, despite vancomycin resistance (Figure 3A). In *E. faecium*, 1.00 μg/mL 3HF enhanced the antibacterial activities of ampicillin, oxytetracycline, streptomycin, and vancomycin (Figure 3B).

### 2.3. 3HF Treatment Dose-Dependently Reduced Bacterial Adherence to T24 Cells

**Figure 4:**
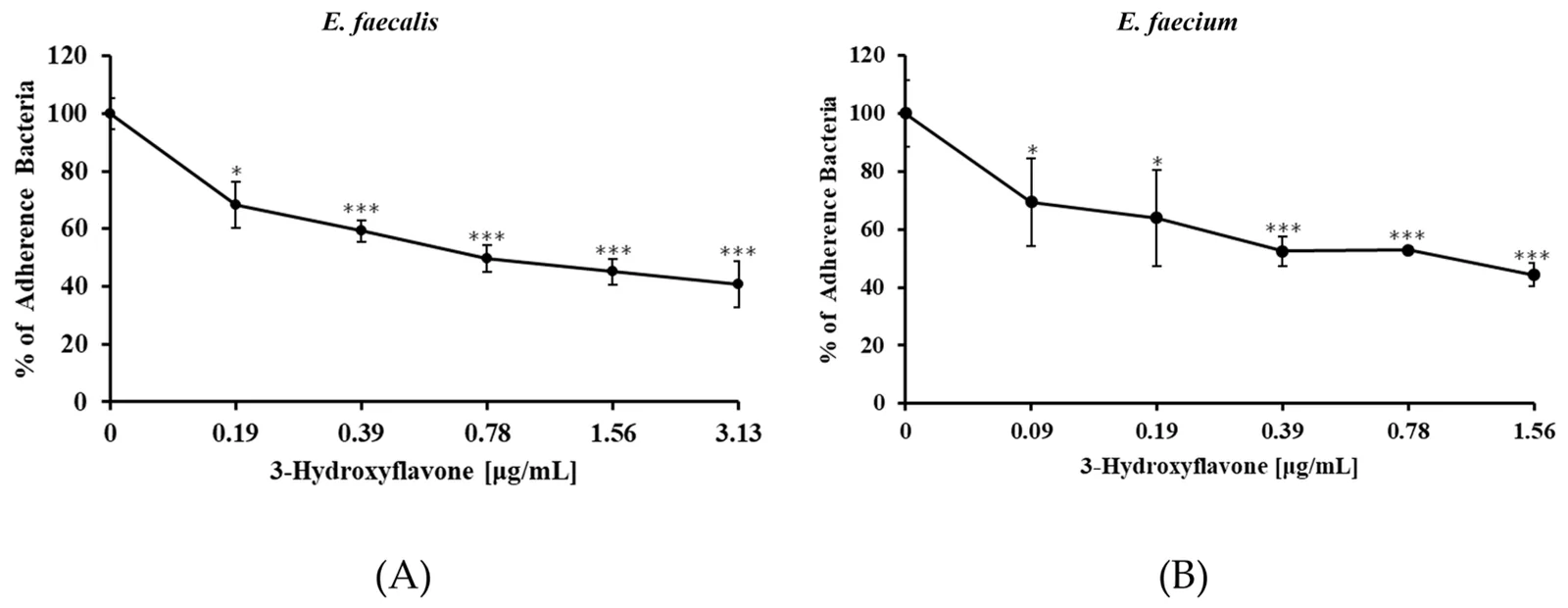
3HF reduces bacterial adherence to T24 host cells in vitro. (**A**) *E. faecalis* and (**B**) *E. faecium*. Data are presented as mean ± SD (n = 3). Statistical significance was evaluated relative to the untreated control (* *p* < 0.05, *** *p* < 0.001).

3HF significantly inhibited biofilm formation at concentrations of 0.01–25 μg/mL. Based on these effective biofilm-inhibitory concentrations, *E. faecalis* was treated with 0.19–3.13 μg/mL 3HF to determine whether the compound could inhibit bacterial adherence to human urinary bladder cancer T24 cells at biologically active doses. Microscopic analysis revealed that 3HF dose-dependently suppressed bacterial adhesion to T24 cells. Furthermore, no morphological signs of cytotoxicity (e.g., cell rounding, detachment, or monolayer disruption) were visually observed in the T24 host cells at these tested concentrations (Supplementary Figure S1). Notably, treatment with 3.13 μg/mL 3HF reduced *E. faecalis* adherence by 59% compared to the untreated control. Similarly, 3HF dose-dependently reduced *E. faecium* adherence to T24 cells, with 1.56 μg/mL treatment decreasing adherence by 56% (Figure 4).

### 2.4. 3HF Did Not Inhibit Bacterial Growth

**Figure 5:**
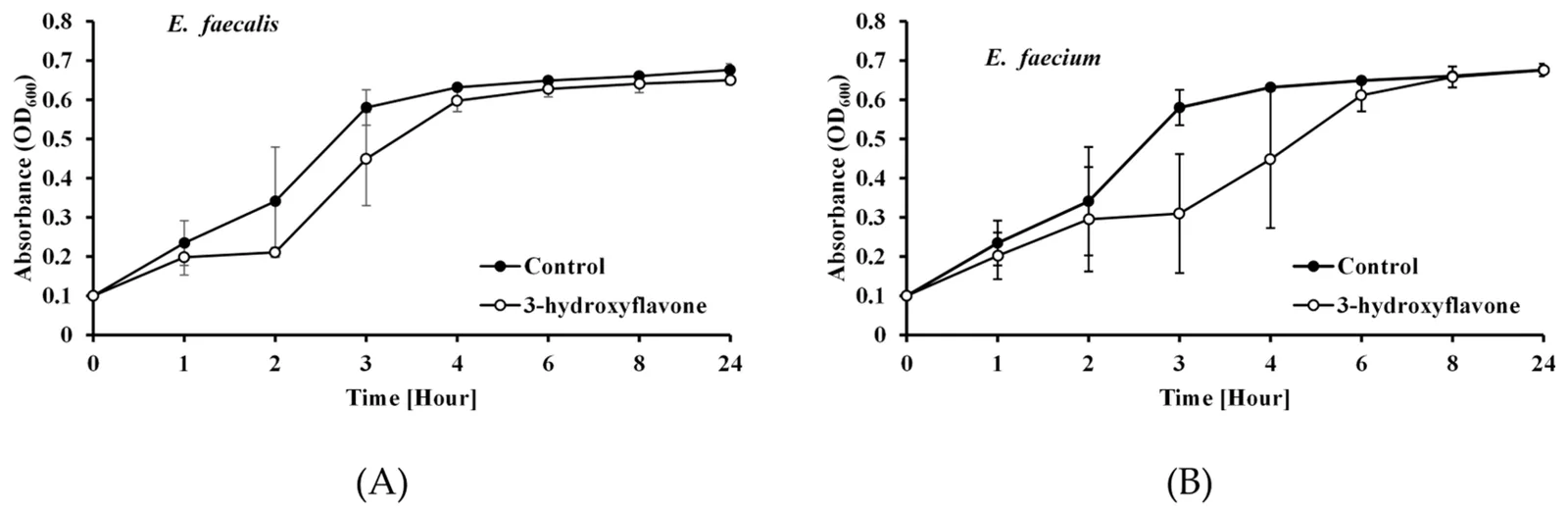
3HF does not inhibit the planktonic growth of (**A**) *E. faecalis* and (**B**) *E. faecium*. Data are presented as mean ± SD (n = 3).

Some antibacterial agents also inhibit biofilm formation, as bacterial killing reduces the number of cells and thereby indirectly limits biofilm development [28]. To determine whether the biofilm-inhibitory effect of 3HF was attributable to its antibacterial activity, 50 μg/mL of 3HF was applied to *E. faecalis* and *E. faecium* for 24 h. 3HF did not affect bacterial growth under these conditions (Figure 5).

### 2.5. 3HF Reduced the Expression of Biofilm Formation-Related Genes

**Figure 6:**
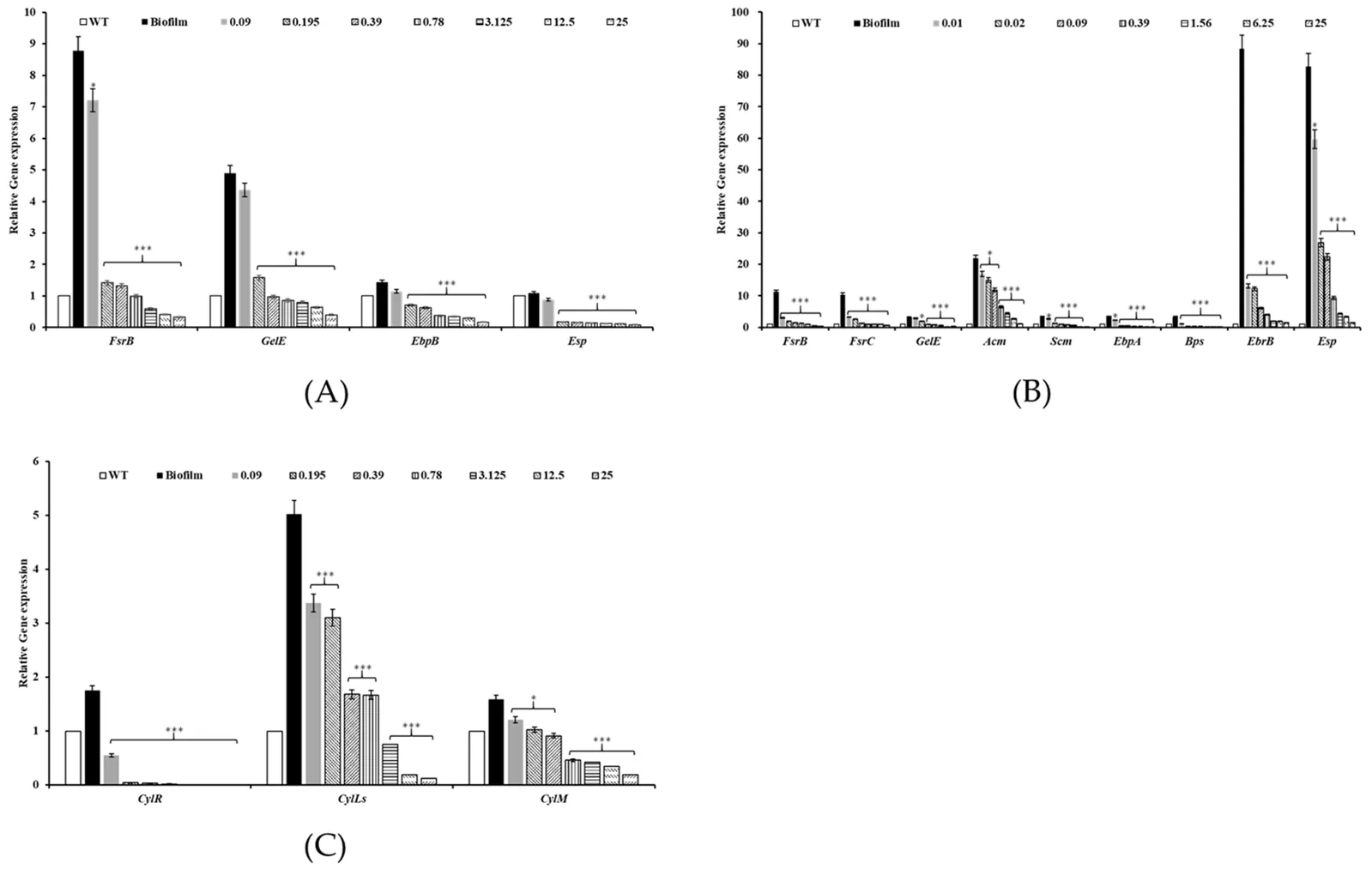
3HF transcriptionally modulates the expression of genes associated with biofilm formation and bacterial virulence. Effect of 3HF on quorum-sensing and biofilm formation genes in (**A**) *E. faecalis* and (**B**) *E. faecium*. (**C**) Effect of 3HF on enterococcal cytolysin synthesis genes. WT represents planktonic growth cells, and Biofilm indicates the untreated negative control. Data are presented as mean ± SD (n = 3). Statistical differences were analyzed compared to the untreated Biofilm control. Data are presented as the mean ± S.D. from three independent experiments (n = 3) (* *p* < 0.05, *** *p* < 0.001).

3HF suppressed the expression of genes involved in biofilm formation and bacterial virulence. To investigate the transcriptional modulation associated with this biofilm inhibition, we examined the expression of genes associated with biofilm-related factors, quorum sensing, and cytolysin systems using qRT-PCR. 3HF dramatically reduced the expression of biofilm-associated genes, including *Fsr* quorum-sensing genes (*FsrB*, *GelE*, *EbpB*, and *Esp*) in *E. faecalis* and (*FsrB*, *FsrC*, *GelE*, *Acm*, *Scm*, *EbpA*, *Bps*, *EbrB*, and *Esp*) in *E. faecium* (Figure 6A,B). In addition, 3HF dose-dependently decreased the expression of genes involved in enterococcal cytolysin synthesis, including *CylR*, *CylLs*, and *CylM* (Figure 6C).

## 3. Discussion

Pathogenic bacteria utilize biofilm formation to increase tolerance to antimicrobial treatment and promote microbial persistence in clinical infections [3,4,5]. In this study, 3HF broadly inhibited biofilm formation across several bacterial species (Figure 2; Table 2) [28,29,30,31,32,33,34,35,36,37,38,39]; however, *E. faecalis* and *E. faecium* demonstrated the highest susceptibility to 3HF treatment. These enterococci are clinically relevant opportunists notorious for their robust biofilm-forming capacity [5,6,7,8,9,10,11,12,13,14] and high levels of intrinsic and acquired resistance to widely used agents, such as tetracycline, ampicillin, and vancomycin [40,41,42]. Consequently, their enterococcal populations within oral and systemic niches are strongly associated with chronic recurrent infections and therapeutic failure.

The screening results of 14 flavonoids (Table 1) highlight the critical role of the 3-hydroxyl substitution in antibiofilm activity. While flavone and other methoxylated or non-hydroxylated derivatives exhibited minimal effects, 3HF demonstrated profound inhibition, expanding upon limited previous reports of antibacterial activity in some hydroxyflavones [17,18,19]. Recent structure–activity relationship (SAR) studies on flavonoids suggest that the 3-OH moiety, particularly when conjugated with the 4-carbonyl group, significantly enhances hydrogen-bonding capacity and electron delocalization. This structural feature likely facilitates stronger binding affinities to specific bacterial target proteins—such as quorum-sensing receptors—compared to structurally similar analogs lacking this functional group.

Our findings should be contextualized within recent advancements in flavonoid research. Many naturally occurring flavonoids, such as quercetin and baicalein, have been reported to inhibit bacterial biofilms primarily by disrupting membrane integrity or chelating essential metal ions [20]. However, our viability data (Figure 5) indicate that 3HF does not exert direct bactericidal effects on planktonic cells. Instead, 3HF acts specifically as an antivirulence agent. By attenuating biofilm formation without inhibiting overall growth, 3HF minimizes the selective survival pressure that typically drives the rapid emergence of drug resistance.

At the molecular level, 3HF significantly downregulated the transcription of the *Fsr* quorum-sensing system (*FsrB*, *FsrC*) and its associated virulence factors (*GelE*, *Esp*). The *Fsr* system is a well-established master regulator of enterococcal biofilm development. Recent studies have demonstrated that inhibiting this network disrupts extracellular polymeric substance (EPS) production and surface adhesion [15,43,44,45]. Consequently, the structural integrity of the biofilm is compromised, which likely explains the enhanced antibiotic susceptibility observed in our study (Figure 3). The breakdown of the protective biofilm barrier allows conventional antibiotics, such as vancomycin, to penetrate more effectively and access the enclosed bacterial cells.

Despite these promising in vitro findings, several limitations of this study must be addressed. First, our study clearly demonstrates the inhibition of initial biofilm formation, but the eradication of pre-existing, mature biofilms was not evaluated. Second, while enhanced antibacterial activity was observed, establishing formal pharmacological synergy requires further validation through specific drug interaction methodologies, such as fractional inhibitory concentration (FIC) index determination via checkerboard assays. Third, the precise molecular targets of 3HF remain unidentified. Given the apparent functional importance of the 3-OH group, future research should incorporate in silico molecular docking analyses to predict and verify the direct binding interactions between 3HF and key enterococcal *Fsr* target proteins. Finally, any clinical or therapeutic applications of 3HF as an antibacterial adjuvant must be rigorously evaluated in in vivo infection models.

## 4. Materials and Methods

### 4.1. Bacterial Strains

All experiments and materials used in this study were conducted as described in our previous publications [13,14].

3-HF was dissolved in DMSO. A solvent control containing the corresponding concentration of DMSO was included in all experiments and showed no effect on bacterial growth or biofilm formation.

The strains used in this study are listed in Table 2, along with the IC_50_ values of 3HF. *E. faecalis*, *E. faecium*, *E. coli*, and *S. aureus* were maintained in tryptic soy broth supplemented with 1% glucose (TSBg). *S. mutans* and *S. sobrinus* were maintained in brain heart infusion (BHI) medium. *P. gingivalis* was maintained in tryptic soy broth supplemented with 10% defibrinated horse blood agar, *P. aeruginosa* was maintained in nutrient agar, and *C. acnes* was maintained in reinforced clostridial agar and incubated at 37 °C [13,14,46,47,48].

### 4.2. Biofilm Formation Assay

Biofilm formation was assessed using the medium indicated for each bacterium in the bacterial strains section. Bacterial cultures were adjusted to an OD_600_ of 0.1, and 200 μL of each suspension was inoculated into 96-well flat-bottom microplate. 3HF (H4280; Sigma Aldrich, Saint Louis, MO, USA) was added at the indicated concentrations, and the plates were incubated for 24 h under appropriate growth conditions. Following incubation, the wells were gently washed with 200 μL phosphate-buffered saline (PBS) to remove non-adherent cells. The remaining biofilms were stained with 100 μL of 1% (*w*/*v*) crystal violet solution (V5265; Sigma-Aldrich) for 30 min at room temperature. Excess stain was removed by washing the wells with 200 μL PBS, and the bound crystal violet was solubilized with 150 μL of 30% (*v*/*v*) acetic acid for 15 min. Biofilm biomass was quantified by measuring the absorbance at 595 nm using a microplate reader (BioTek Instruments, Seoul, Korea), as described previously [13,14,46,47,48]. The half-maximal inhibitory concentration (IC_50_) values for biofilm formation were calculated using Microsoft Excel software.

### 4.3. Enhanced Antibacterial Effects of 3HF Combined with Commercial Antibiotics

The enhanced antibacterial effects of the combination of 3HF and antibiotics against biofilm-associated bacteria were evaluated using a colony-forming unit (CFU) plate counting assay. Biofilms of *E. faecium* and *E. faecalis* were prepared as described above. Briefly, 1 mL of the bacterial suspension was treated with ampicillin (A9518; Sigma Aldrich: 6.25 μg/mL), vancomycin (V0045000; Sigma Aldrich: 100 μg/mL), oxytetracycline (O5875; Sigma Aldrich: 3.125 μg/mL), or streptomycin (SBR00068; Sigma Aldrich: 100 μg/mL) in the presence of the indicated concentrations of 3HF. Antibiotic treatment alone (without 3HF) was used as a control. Following treatment, biofilm-associated bacteria were recovered, serially diluted, and plated on Luria–Bertani (LB) agar plates. After incubation, viable bacterial counts were determined by enumerating colony-forming units (CFUs), as previously described [13,14,47].

### 4.4. Growth Assay

*E. faecalis* and *E. faecium* were cultured in TSBg and adjusted to an optical density at 600 nm (OD_600_) of 0.1. 3HF was added to the cultures at a final concentration of 50 μg/mL, and the cells were incubated at 37 °C. Bacterial growth was monitored by measuring the optical density at 600 nm at the indicated time points using a microplate reader [13,14].

### 4.5. Cell Adhesion Assay and Morphological Observation

Human bladder carcinoma T24 cells were cultured in RPMI-1640 medium supplemented with 10% fetal bovine serum (FBS) and 1% penicillin–streptomycin at 37 °C in a humidified 5% CO_2_ atmosphere. T24 cells were seeded into culture plates and grown to a confluent monolayer. Prior to infection, the cells were washed with phosphate-buffered saline (PBS). For the adhesion assay, bacterial suspensions were added to the T24 cells in the presence or absence of 3HF and incubated for 24 h at 37 °C. Following incubation, the cells were gently washed three times with PBS to remove non-adherent bacteria.

To quantify adherent bacteria, the host cells were lysed using 0.1% Triton X-100, and the lysates were serially diluted and plated onto agar plates for colony counting. For morphological observation and cytotoxicity assessment, the washed cells and adherent bacteria were fixed, stained with 0.1% crystal violet, and photographed using an inverted optical microscope. Cytotoxicity was evaluated by observing any morphological changes or monolayer disruptions in the T24 cells under the microscope.

### 4.6. Quantitative RT-PCR Analysis

The bacterial culture conditions and 3HF treatment were the same as those used in the biofilm inhibition assay. Total RNA was extracted using TRIzol reagent (Life Technologies, Thermo Fisher Scientific, Waltham, MA, USA) according to the manufacturer’s instructions. The concentration and purity of the extracted RNA were confirmed by measuring the A260/A280 ratio using a spectrophotometer (e.g., NanoDrop; Thermo Fisher Scientific, Waltham, MA, USA). First-strand cDNA was synthesized from 1 μg of total RNA using a reverse transcription kit (NanoHelix, Daejeon, Korea). Quantitative real-time PCR (qRT-PCR) was performed using 2× SYBR Green qPCR Master Mix (CellSafe, Yongin-si, Korea). The primer sequences used in this study are listed in Table 3. Primer amplification efficiencies were evaluated and confirmed to be within the acceptable range. The 23S rRNA and tufA genes were validated as stable endogenous reference genes for normalization under our experimental conditions. Furthermore, no-template controls (NTC) were included in each PCR run to rule out DNA contamination. Relative gene expression levels were calculated using the 2^−ΔΔCt^ method [13,14,22,36,37,38,39,40,41,42,43,44,45,46,47,48].

### 4.7. Statistical Analysis

All experiments were performed in independent biological triplicates (n = 3) for each condition, and the data are presented as mean ± standard deviation. Statistical significance was determined using one-way ANOVA followed by Tukey’s post hoc test, with *p* < 0.05 considered statistically significant. Statistical annotations are denoted as * *p* < 0.05 and *** *p* < 0.001.

## 5. Conclusions

In conclusion, the present in vitro study demonstrates that 3HF effectively inhibits biofilm formation in several bacterial species, notably *E. faecalis* and *E. faecium*, without directly inhibiting planktonic bacterial growth. Furthermore, 3HF downregulates the transcription of quorum-sensing and virulence-associated genes and significantly enhances the antibacterial activity of conventional antibiotics against these resistant strains. These findings suggest that 3HF has the potential to serve as an antibacterial adjuvant for controlling biofilm-associated infections. However, further studies, including in vivo validation, are necessary to determine its clinical applicability.

## Figures and Tables

**Table 1:** The inhibitory effect of flavonoids at 100 µg/mL concentration on *E. faecalis* biofilm formation. Data are presented as mean values of independent biological replicates (n = 3).

Compounds	Inhibition ^1^
Flavone	2.01%
3-Hydroxyflavone	89.02%
5-Hydroxyflavone	3.08%
7-Hydroxyflavone	1.17%
2′-Methoxyflavone	1.82%
5,7-Dihydroxyflavone	5.32%
7,4′-dimethyl-3-hydroxyflavone	3.78%
6,3′-dimethyl-3-hydroxyflavone	4.22%
4′,5,7-trihydroxyflavone	3.71%
3,3′,4′,7-Tetrahydroxyflavone	1.77%
3,3′,4′,5,7-Pentahydroxyflavone	12.88%
4′,5,7-Trihydroxyflavanone	4.42%
3,4′,5,7-Tetrahydroxyflavone	5.39%

^1^ The experiment was performed in independent triplicates, and the results are presented as mean values.

**Table 2:** Bacterial species used in this study and their IC_50_ values for biofilm formation inhibition.

Strain	Strain Number	IC_50_ (Half Maximal Inhibitory Concentration; μg/mL)	Source
*E. faecalis*	CCARM 5511	1.025	Purchased from KACC (Korean Agricultural Culture Collection; Wanju-gun, Republic of Korea), CCARM (Culture Collection of Antimicrobial Resistant Microbes; Seoul, Republic of Korea), or KCTC (Korean Collection for Type Cultures; Jeongeup-si, Republic of Korea)
*E. faecium*	KACC11954	0.19	
*E. coli*	KACC11598	10.88	
*S. mutans*	KACC16833	>50	
*S. sobrinus*	CCARM3506	5.49	
*S. aureus*	KCTC5809	1.21	
*P. aeruginosa*	KACC14021	22.1	
*C. acnes*	CCARM9009	0.132	
*P. gingivalis*	KCTC5352	>50	

**Table 3:** Gene-specific primers were used for quantitative real-time PCR.

Genes	Primer Sequence: 5′ to 3′	Function	Reference
For *E. faecalis*			
*FsrB*	F: TGCTCAAAAAGCAAAGCCTTATAA R: GATGACGAGACCGTAGAGTATTACTGAA	Efae regulator	[46]
*GelE*	F: CGGAACATACTCAACGTTTGA R: TGGATTAGATGCACCCGAAAT	Gelatinase	[46]
*EbpB*	F: CGTACAGGAGGCAAGTCTTT R: AGGTATTCCCCGCTTGATTT	Biofilm-associated pili	[46]
*Esp*	F: GCATCAGTATTAGTTGGT R: TTCCTTGTAACACATCAC	Biofilm formation	[46]
*CylR1*	F: TTTATTTTTATTGGATATCATTCTGTAGTC R: TTCGCTCATCTTTTTTGAATACAG	Cytolysin regulatory	[46]
*CylLs*	F: CTGTTGCGGCGACAGCT R: CCACCAACCCAGCCACAA	Cytolysin toxin	[46]
*CylM*	F: TCGGACACGGTATATATAGCTATGT R: TCTACTAGTGTACTTTGATTACCATAATAATT	Cytolysin toxin	[46]
For *E. faecium*			
*FsrB*	F: TGCTCAAAAAGCAAAGCCTTATAA R: GATGACGAGACCGTAGAGTATTACTGAA	Efae regulator	[49]
*FsrC*	F: GCTTATTTGGAAGAACAACGTATCAA R: CGAAACATCGCTAGCTCTTCGT	Efae regulator	[49]
*GelE*	F: CGGAACATACTGCCGGTTTAGA R: TGGATTAGATGCCACCCGAAAT	Gelatinase	[49]
*Acm*	F: TCAGCAGTAATGTCACTTCGTTG R: GAATAGGCTGTTCATCTGCTCG	Gelatinase	[22]
*Scm*	F: CTAACTGGTAACTATGGCTTGT R: GTCCGTGCTGTCACTTGT	Gelatinase	[22]
*EbpA*	F: ACCAAGCCAGACGAAATAGAAGAAG R: ATTGTTTTGGTCAGGTGCATCATAGA	Biofilm-associated pili	[50]
*Bps*	F: TATCAGCAACAAGCGGTCAA R: AATCCTGCCCTTTTTCGATT	Biofilm formation	[50]
*EbrB*	F: TGAGGGATTCTGGGATTGTTT R: GCCGATGAATTGAACGACAGA	Biofilm Regulator	[21]
*Esp*	F: CCACGAGTTAGAGGGAACAG R: TTGGAGCCCCATCTTTTTCA	Biofilm formation	[21]
*tufA*	F: TACACGCCACTACGCTCAC R: AGCTCCGTCCATTTGAGCAG	Housekeeping gene	[21]
23s RNA	F: CCTATCGGCCTCGGCTTAG R: AGCGAAAGACAGGTGAGAATCC	Housekeeping gene	[46]

## Data Availability

Data are contained within the article.

## Supplementary Materials

The following supporting information can be downloaded at: https://www.mdpi.com/article/10.3390/antibiotics15090925/s1, Figure S1: Morphological observation of T24 host cells following bacterial infection and 3HF treatment.

## Abbreviations

The following abbreviations are used in this manuscript:

3HF	3-Hydroxyflavone
IC_50_	Half Maximal Inhibitory Concentration
KACC	Korean Agricultural Culture Collection
CCARM	Culture Collection of Antimicrobial Resistant Microbes
KCTC	Korean Collection for Type Cultures
TSB	Tryptic soy broth
LB	Luria–Bertani Medium
PBS	Phosphate-buffered saline
CFU	Colony-forming unit
