# Supplementary material for: 3-Hydroxyflavone Inhibits Biofilm Formation and Enhances Antibiotic Activity Against Enterococcus faecalis and Enterococcus faecium
Source: Antibiotics (Basel). 2026 Sep 18;15(9):925. doi: 10.3390/antibiotics15090925 (PMC13603653; doi:10.3390/antibiotics15090925)
Supplement: Supplementary file 1 [file antibiotics-15-00925-s001.zip › antibiotics-4529048-supplementary.pdf]

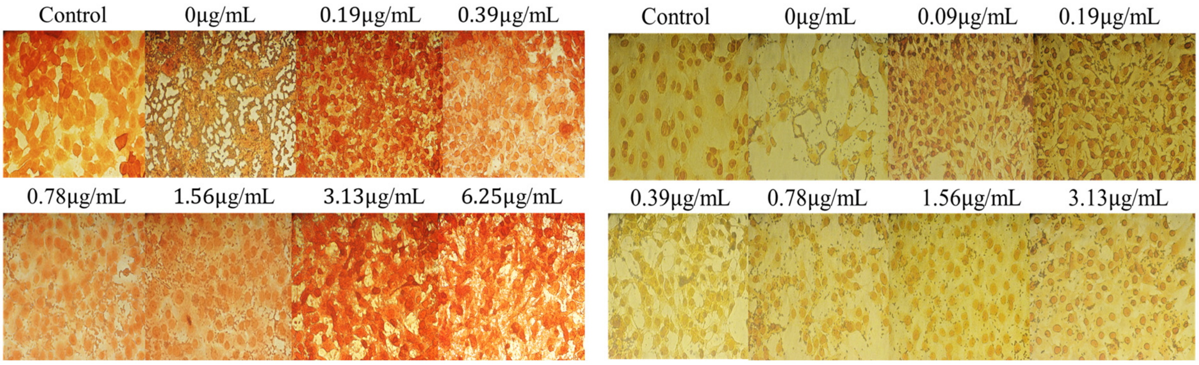

Supplementary Figure S1. Morphological observation of T24 host cells following bacterial infection and 3HF treatment. (Left) T24 cells infected with E. faecalis. (Right) T24 cells infected with E. faecium. 'Control' represents uninfected, healthy T24 cells. '0 µg/mL' represents T24 cells infected with bacteria but without 3HF treatment, showing expected infection-induced cytopathic effects. The intact monolayer and cell shapes in the 3HF-treated groups indicate that no significant morphological signs of cytotoxicity were observed in the T24 cells at the tested concentrations, supporting its safety and protective effect for the in vitro adhesion assays.
